# When can coronary computed tomography angiography in patients with calcified plaque be accurate?

**DOI:** 10.3389/fcvm.2025.1570517

**Published:** 2025-09-22

**Authors:** Qisheng Ran, Diyou Chen, Huiru Zhang, Letian Zhang

**Affiliations:** ^1^ Department of Radiology, Daping Hospital, Army Medical University, Chongqing, China; ^2^ Chongqing Clinical Research Centre of Imaging and Nuclear Medicine, Chongqing, China; ^3^ Chongqing Municipal Key Clinical Specialty, Chongqing, China; ^4^ Department of Gastroenterology, Daping Hospital, Army Medical University, Chongqing, China

**Keywords:** computed tomography angiography, invasive coronary angiography, calcified plaque, coronary artery disease, coronary stenosis

## Abstract

**Aim:**

To identify a new method to indicate when coronary computed tomography angiography (CCTA) in patients with calcified plaque can be accurate.

**Methods:**

Prospective analysis on 105 cases of coronary artery stenosis with calcified plaque underwent both CCTA and invasive coronary angiography (ICA). The Hounsfield unit (Hu) values of calcified plaque and adjacent blood were measured, and then the ratio (R*_Hu_*) was subsequently calculated. The ICA data served as the gold standard for defining obstructive stenosis (≥ 50%) and were utilized to create a two-dimensional receiver operating characteristic (ROC) curve. The cut-off value was employed to categorize the CCTA data. Additionally, a Bland-Altman plot was used to analyze discrepancies in stenosis degree detection between CCTA and ICA. An *in vitro* experiment was designed to assess the practicability of R*_Hu_*.

**Results:**

The R*_Hu_* was correlated with the concordance of CCTA and ICA for stenosis evaluation (*r* = 0.509, *p* < 0.001). ROC analysis suggested a cut-off value of 0.36. The Bland-Altman plot indicated that stenosis evaluation by CCTA demonstrates good concordance when R*_Hu_* exceeds 0.36; however, significant bias occurs when R*_Hu_* is below 0.36 in comparison to ICA. *In vitro* experiments confirmed that the R*_Hu_* parameter can be easily adjusted to enhance the accuracy of CCTA. In validation experiments, the R*_Hu_* achieved a prediction accuracy of 74.0%.

**Conclusion:**

Our study suggests that the accuracy of detection of stenosis with CCTA in calcified vessels is related to the difference in Hu values between calcified plaques and blood.

## 1 Introduction

Coronary artery disease (CAD) is one of the leading causes of mortality worldwide (1). The pathological nature of CAD involves a progressive increase in coronary plaque size, which can potentially lead to plaque rupture and coronary artery thrombosis (2). Many studies have concluded that approximately 50%–70% of coronary artery plaques are calcified in populations that are either asymptomatic or suspected CAD (3–5). The gold standard for CAD diagnosis remains conventional invasive coronary angiography (ICA), however, its invasive nature and insensitivity to calcified plaques and small non-calcified plaques in the vessel wall limit its ability to detect stenosis among populations at low or intermediate risk for this condition, such as those with atherosclerotic disease (6). Multi-detector computed tomography (MDCT) is an emerging imaging modality for the first-line detection of CAD due to its fine spatial and temporal resolution. Furthermore, MDCT can non-invasively image both the lumen and wall of coronary artery vessels, distinguishing between calcified, non-calcified, and mixed plaques, as well as determining the degree of luminal stenosis (7–11).

Currently, one of the primary limitations of coronary computed tomography angiography (CCTA) is its inaccuracy in assessing calcified lesions (12–14). Beam-hardening and blooming artifacts have led to both overestimation and paradoxical underestimation of the severity degree of calcification-related stenosis (15). The beam-hardening effect arises from divergence absorption of low-energy photons as a monoenergetic beam passes through a given material (16). The resulting “filter” effect produce a higher distribution of high-energy (or “harder”) photons, which raises the average energy of the beam and leads to low-attenuation artifacts (17). The majority of false-positive results have been observed in highly calcified segments, primarily due to partial volume effects. To improve diagnostic precision of CCTA, a comprehensive understanding of the relationship between the intrinsic features of calcium plaques and the severity of coronary vessel lumen distortion is essential. Beam-hardening correction (BHC) algorithms, the most commonly used methods for correcting beam-hardening artifacts over the past few decades, have effectively calibrated these artifacts caused by tissues with substantial high-attenuation sources (e.g., bone) (18). Filtered back projection (FBP) algorithms can reduce blooming artifacts but often at the expense of image quality or by increasing radiation exposure through improved spatial resolution achieved via thinner collimated section widths, reconstruction thickness, and higher-resolution sharper reconstruction algorithms (19). Iterative reconstruction techniques can decouple spatial resolution and noise, offering a potential tool to reduce blooming artifacts. However, the effectiveness of iterative reconstruction largely depends on the image acquisition technique employed [e.g., prospective electrocardiographic (ECG) triggering vs. retrospective ECG gating] (20).

Few studies have explored the use of non-algorithmic methods to improve the accuracy of CCTA assessments of calcified lesions. Consequently, we designed this study to enhance clinical relevance, with a primary focus on exploring the relationship between the Hounsfield unit (Hu) values of calcified plaque and adjacent blood, as well as the accuracy of CCTA in assessing coronary lumen stenosis.

## 2 Methods

### 2.1 Patients

This prospective study was designed to evaluate the accuracy of CCTA for the detection of coronary calcification stenosis in symptomatic patients to aid in the clinical decision-making of ICA. The study comprised three components: an evaluation experiment, an *in vitro* experiment, and validation experiments. All patients had ICA scheduled based on the evaluation of cardiologists, with indications including suspected CAD manifesting as chest pain or abnormal or equivocal nuclear stress test results over a three-year period. Patients with soft plaque were excluded from this study. The total cohort was 143 patients, divided into the evaluation cohort (*n* = 105; 55 men, 50 women; mean age, 64.8 ± 9.36 years) and the validation cohort (*n* = 38; 28 men, 10 women; mean age, 68.1 ± 6.94 years), all having an Agatston score greater than 100. The study was approved by the institutional review board of our Medical University and was conducted in compliance with Health Insurance Portability and Accountability Act regulations. All participating patients provided informed consent for their involvement in the study, and all experimental protocols adhered to the Declaration of Helsinki.

### 2.2 CCTA acquisition

A non-contrast-enhanced calcium score scan (detector collimation = 64 × 1.5 mm, 120 kV, ECG gating, slice thickness = 3 mm, medium smooth kernel B35f) was performed for the Agatston Score calculations (21). Then, CCTA examinations were conducted on 256 MDCT units (iCT, Brilliance, Philips Healthcare) and postprocessed on workstations (Brilliance, Philips Healthcare). After the leads for the ECG recording were positioned and adequate peripheral venous access was established, the heart rate of the patient was monitored. Betaloc (25–50 mg) was administered intravenously to achieve a target heart rate of 70 beats per minute (bpm). An automated bolus-tracking scan was performed to initiate the CCTA sequence. Repetitive monitoring scans were conducted at a single slice level during respiration at 50 mA with a scan time duration of 0.35 s. The monitoring scans commenced after a delay of 6 s and were repeated every 1.5 s during quiet breathing. When the contrast enhancement threshold of 100 Hu was reached within the predefined slice (i.e., lumen of the descending aorta), the CCTA scans were initiated. Approximately 5 s after exceeding the threshold level, allowing time for repositioning the table and providing breathing instructions to the patient, the arterial phase scan was commenced. A contrast bolus of iohexol-350 (350 mg I/ml) was injected at a ﬂow rate of 4–6 ml/s through an 18–20-gauge intravenous antecubital catheter by using a power injector (Ulrich, Germany). The total dose of iohexol-350 administered was approximately 0.8–1.0 ml/kg of body weight, which is well below the FDA-recommended dose for elderly patients with pre-existing renal disease.

The CCTA scan parameters were as follows: detector collimation of 1 × 128 × 0.625 mm; gantry rotation time of 270 ms; and a tube current-time product, ranging from 250 to 1,000 mAs per rotation. The tube potential was set at 120 kV for patients with a body mass index (BMI) of 25 kg/m^2^ or greater, while it was adjusted to 100 kV for patients with a BMI of less than 25 kg/m^2^. Data acquisition occurred in the craniocaudal direction, extending from above the origin of the coronary arteries to below the diaphragm dome. The effective radiation dose was calculated by multiplying the dose-length product by a chest-specific conversion coefficient (k = 0.014 mSv/Gy/cm).

The measurement steps for coronary artery stenosis were detailed in the supplementary materials and Figure S1. Based on the routine scanning practices of our department, we found that the difference in Hu values between blood and calcified plaques within a single voxel may be associated with the overestimation of coronal stenosis. Consequently, we assessed the ratio of the Hu value of blood to that of calcified plaque (R*_Hu_*) and measured the degree of stenosis as observed on CCTA. We utilized mean HU values. Region of interest (ROIs) for plaque were placed entirely within the calcified component, ensuring that the high-contrast edge was avoided by an inward margin of at least 0.3–0.5 mm. Three small circular ROIs, each measuring between 0.5 and 1.0 mm², were analyzed across three consecutive slices, and the results were averaged. Blood ROIs were positioned in the central lumen of the same segment, or in the immediately proximal segment when necessary, maintaining a distance of at least 1 mm from the wall to prevent partial volume effects and blooming artifacts. These values were also averaged across three slices (Supplementary Figure S2). The ratio of Hu value between the blood and plaque (R*_Hu_*) was calculated using the equation provided below.

$$R_{Hu}=\frac{Hu_{blood}}{Hu_{calification}}$$

### 2.3 Coronary angiography

ICA was performed using the standard Judkins method, and the images were reviewed by senior cardiologists (possessing over 10 years of experience). The coronary arteries were classified according to the American College of Cardiology and the American Heart Association (ACC/AHA) Classification system (22). There were no discrepancies among the interpretations made by the radiologists, radiographers, and cardiologists involved in the studies. Segments were categorized as either nonobstructive disease (characterized by luminal irregularities or < 50% diameter stenosis) or obstructive disease (≥ 50% diameter stenosis). A stenosis of fifty percent was established as the benchmark for determining the necessity of invasive treatments (23). The median interval between CCTA and ICA was 5 days [IQR (3–7)]. Specifically, 28% of patients underwent ICA within 3 days of CCTA, 50% within 4–7 days, and 22% within 8–14 days.

### 2.4 Data postprocessing

For the CCTA data, reconstructions and stenosis evaluations were conducted by two experienced radiologists, each possessing over 10 years of experience. This process involved vessel segmentation and tracking using the Comprehensive Cardiac Analysis software package, from Philips Healthcare for curved multiplanar reformation (MPR) image reconstruction, as well as straightening each vessel to obtain long-axis views. Then, we moved the mouse cursor along the entire length of the vessel to acquire cross-sectional images. Two radiographers, also with over 10 years of experience, performed the measurements independently, primarily based on the appearance of the arterial lesions in the cross-sectional views. The curved MPR images and straightened long-axis views served as primary references when the quality of the cross-sectional images was deemed too poor or suboptimal for measurement purposes. Additionally, one cardiologist, with more than 10 years of experience, reviewed ICA images to measure the stenosis diameters. The criterion for “obstructive” stenosis was defined as more than 50% of the lumen diameter being consistently occupied by calcified coronary artery plaque.

The degree of stenosis, expressed as a percentage of diameter, was evaluated using both CCTA and ICA. The severity of coronary stenosis was quantified by measuring the diameter at maximum stenosis and the reference diameter for all stenosis. The percentage of stenosis was calculated using the following formula (24):

$$\text{\%}Stenosis=\frac{Diameter_{\;poststenotic\;vessel}-Diameter_{max\;stenosis}}{Diameter_{\;poststenotic\;vessel}}$$

The difference between CCTA and ICA on stenosis evaluation was expressed as a ratio of the degree of stenosis.

For the validation experiment, we generated cumulative histograms for the R*_Hu_* in patients whose coronary results were either consistent or inconsistent with ICA results, respectively. Additionally, the prediction accuracy of R*_Hu_* in the validation cohort was determined using the specified formula (25):

$$ACC=\frac{TP+TN}{TP+TN+FN+FP}$$

### 2.5 *In vitro* experiment

*In vitro* experiments were introduced to testify whether the *R_Hu_* parameter can effectively adjust to refine the accuracy of CCTA in measuring the degree of stenosis. The phantom for the *in vitro* experiment was designed to simulate the intussusception of two tubes with differing diameters. The inner tube, with a diameter of 2 mm, was filled with three different concentrations (i.e., 70 wt.%, 60 wt.%, 50 wt.%) of calcium solution, while the outer tuber with a diameter of 20 mm, was filled with a contrast medium (iohexol-350) dissolved in normal saline at three different dilution ratios to obtain varying CT numbers (i.e., 530 Hu, 360 Hu, 250 Hu).

### 2.6 Statistical analysis

The degree of stenosis was evaluated using both CCTA and ICA. Statistical analyses were conducted with SPSS statistical software (SPSS 12.0; SPSS, Chicago, IL). Lesions were classified as obstructive (≥ 50% luminal diameter narrowing) or nonobstructive (< 50% luminal diameter narrowing). ICA data served as the gold standard for true stenosis, facilitating the creation of two-dimensional receiver operating characteristic (ROC) curves. The Strandness criteria for ICA were employed in generating these ROC curves to categorize the CCTA data into binary outcomes (yes/no), allowing for the assessment of diagnostic accuracy regarding stenosis. *P* values less than 0.05 were deemed significant. Interobserver agreement for subjective image quality was quantified using intraclass correlation coefficient (ICC) statistics. Correlations were established using either Kendall's tau or Pearson correlation methods. The diagnostic accuracy for detecting severe stenosis (≥ 50%) was calculated with cardiac catheterization as the reference standard. A ROC curve analysis was performed to determine the cut-off ratio of the R*_Hu_* between blood and plaque. A Bland-Altman plot was utilized to assess the agreement between CCTA and ICA across different R*_Hu_* intervals. Additional model specifications and extended statistical methods for incremental analysis, including net reclassification index (NRI), integrated discrimination improvement (IDI), decision curve analysis (DCA), and calibration, were detailed in the supplementary materials.

## 3 Results

A total of 143 patients met the inclusion criteria, and both CCTA and ICA were successfully performed, yielding diagnostic-quality images. Patient demographics and CCTA characteristics are detailed in Table 1.

**Table 1:** Patient demographics and scan parameters.

Parameters	Evaluation cohort (*n* = 105)	Validation cohort (*n* = 38)
Age(y)	64.8 ± 9.36	68.1 ± 6.94
Male to female ratio	55: 50	28: 10
Height (cm)	161.4 ± 12.4	162.3 ± 11.8
Weight (kg)	63.6 ± 10.3	62.3 ± 9.71
Body mass index (kg/m^2^)	32.3 ± 6.1	31.7 ± 7.3
Heart rate (beats per minute)	72.3 ± 15.2	71.6 ± 18.2
Agatston score	729 ± 303	736 ± 451
Tube current–time product (mAs)	250–300	250–300
Effective dose (mSv)		
Retrospective ECG gated (*n* = 73)	10.9 ± 5.2	11.7 ± 6.9
Prospective ECG triggered (*n* = 70)	3.2 ± 1.8	3.8 ± 2.1
CT dose index volume (mGy)	28.3 ± 17.5	30.7 ± 19.4
Dose-length product (mGy·cm)	451 ± 361	479 ± 392
Pitch	0.2	0.2
No. of mixed plaques	48	15
No. of calcified plaques	57	13

ECG, electrocardiographic; CT, computed tomography.

### 3.1 Relationship of the Hu value difference between blood and calcified plaque

**Figure 1:**
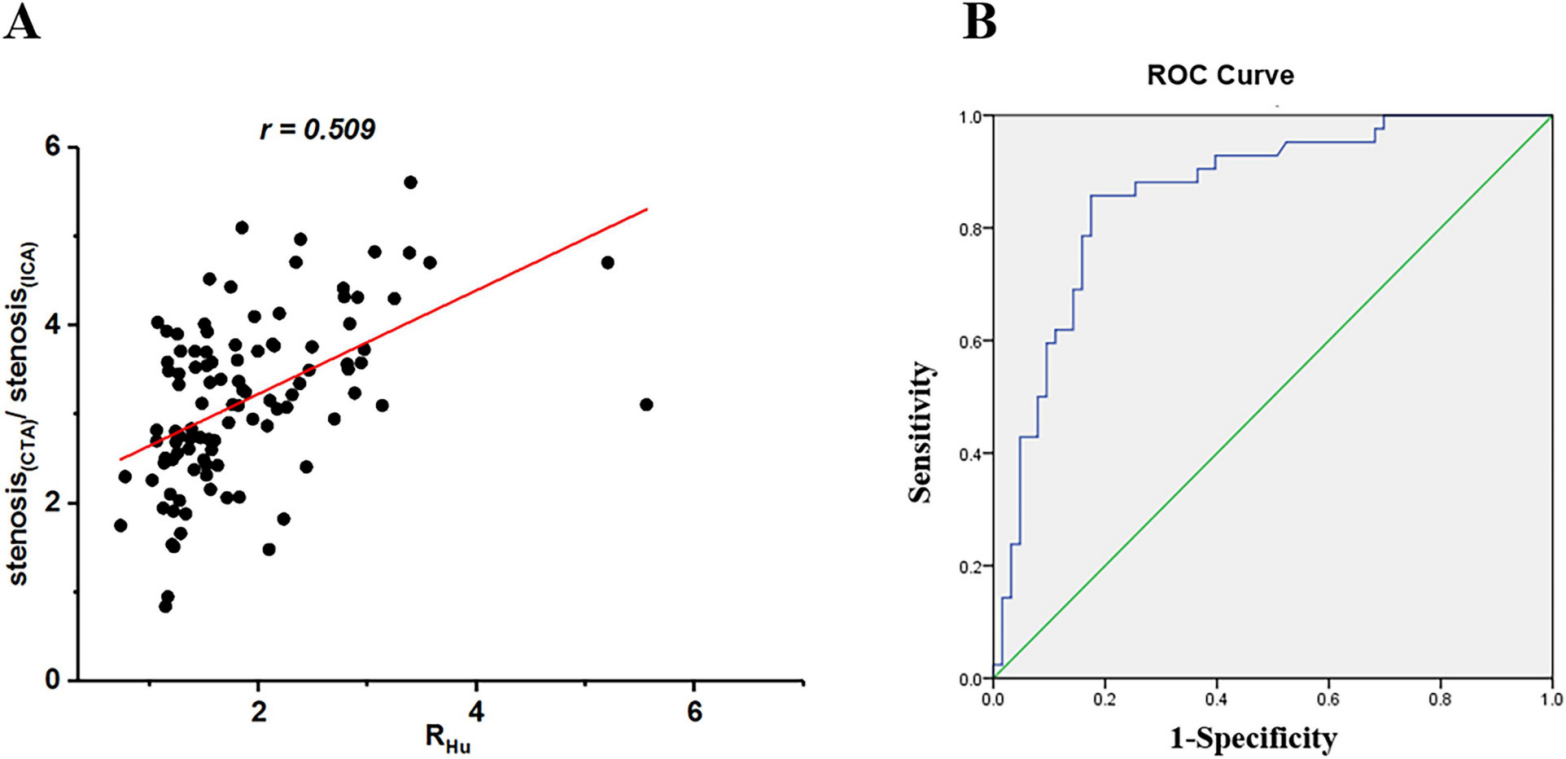
Scatterplots of relationship between ratio of stenosis assessed by coronary CTA versus ICA and Hu values between calcified plaque and blood (R*_Hu_*) **(A)** ROC curve analysis was obtained to predictive value of *R_Hu_* for coronary CTA to detect stenosis degree and determine the cutoff of ratio of *R_Hu_* **(B)** CTA, computed tomography angiography; ICA, invasive coronary angiography; ROC, receiver operating characteristic; Hu, hounsfield units; R*_Hu_*, ratio of HU values between calcified plaque and blood.

The inter-reader agreement for R*_Hu_* classification among radiologists and cardiologists was k = 0.83 and 0.85, respectively. The *R_Hu_* demonstrated a strong correlation with the concordance of CCTA and ICA for stenosis evaluation (*r* = 0.509, *p* < 0.001, 95% CI). ROC curve analysis revealed a significant area under curve (AUC) for the Hu values ratio between the calcified plaque and blood, indicating its effectiveness in assessing the concordance of CCTA from ICA (Figure 1). The AUC and cut-off values were 0.789 and 0.36, respectively. The sensitivity and specificity were recorded at 86.13% and 82.14%, respectively, while the negative predictive value (NPV) and positive predictive value (PPV) were 79.8% and 87.8%, respectively. The NRI was 0.5, and the IDI was 0.248. The DCA (Supplementary Figure S3) demonstrated that the model incorporating R*_Hu_* consistently provided a higher net benefit than CCTA alone across high threshold probabilities (> 90%). Calibration analysis (Supplementary Figure S4) revealed a good agreement between predicted and observed risks.

### 3.2 Diagnostic accuracy of CCTA in calcified plaques at R_*Hu*_ < 0.36 vs. R_*Hu*_ > 0.36

**Figure 2:**
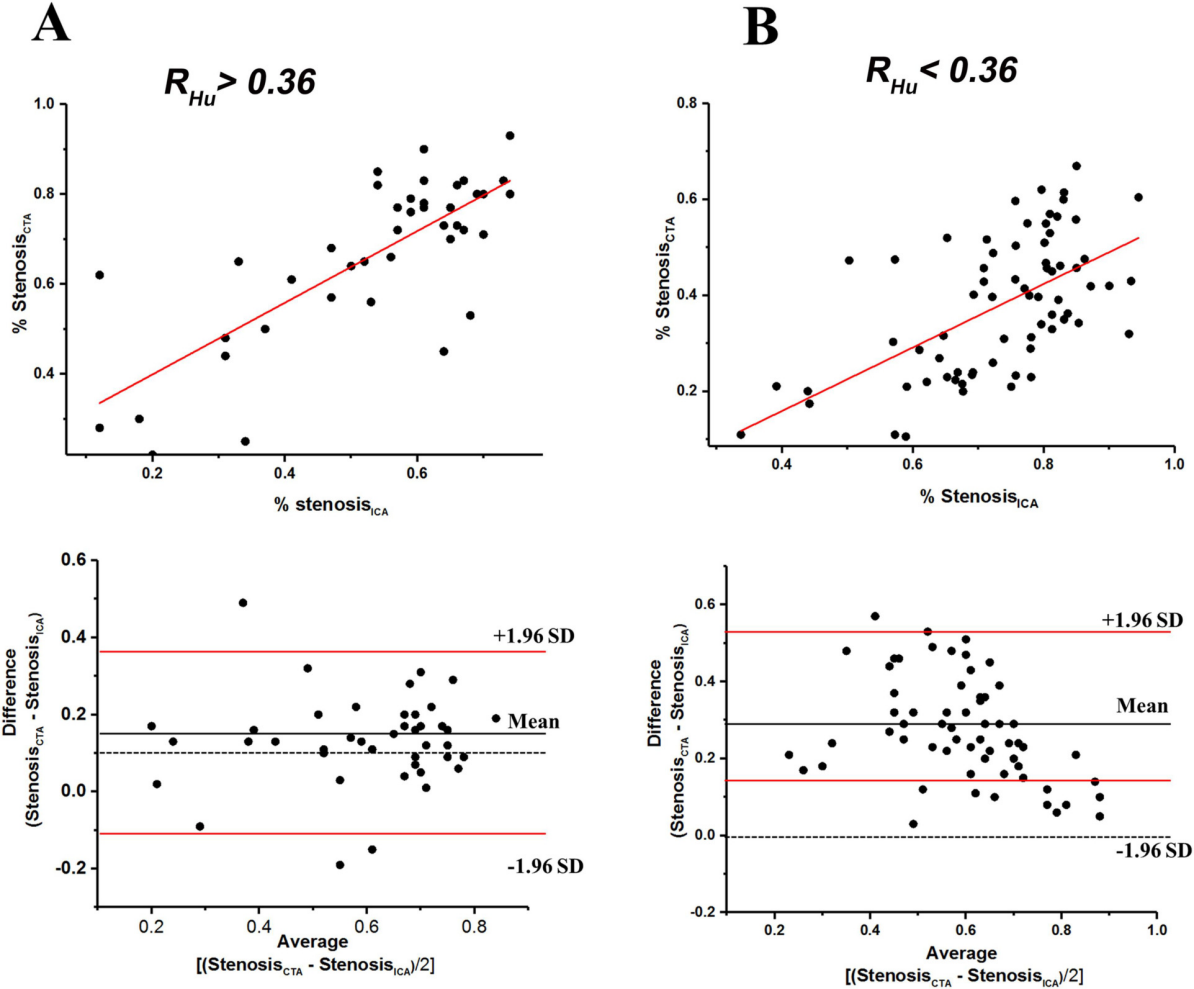
Scatterplots of relationship between stenosis degree (%) measured with coronary CTA and ICA, and bland-altman plots of relative (%) difference between stenosis degree (%) as assessed by coronary CTA versus ICA (reference) within *R_Hu_* > 0.36 **(A)**, and *R_Hu_* < 0.36 **(B)** within the scatterplots the red lines represent fitted regression lines at scatterplots and represent 95% confidence intervals at bland-altman plots. CTA, computed tomography angiography; ICA, invasive coronary angiography; Hu, Hounsfield units; R*_Hu_*, ratio of HU values between calcified plaque and blood; SD, standard deviation.

The diagnostic performance within calcified of CCTA vs. ICA cross sections is illustrated in Figure 2. The ratios of stenosis degree measured by CCTA compared to ICA were significantly different under the *R_Hu_* > 0.36 condition vs. the *R_Hu_* < 0.36 condition [1.37 [0.7–2.48] vs. 1.86 [1.07–5.56], *p* = 0.02]. The degree of stenosis measured by CCTA showed a stronger correlation (Kendall's tau = 0.512; 95% CI) with ICA in the R*_Hu_* > 0.36 condition than in the *R_Hu_* < 0.36 condition (Kendall's tau = 0.463; 95% CI). The Bland-Altman plot indicated that the stenosis evaluation by CCTA had good concordance under the *R_Hu_* > 0.36 condition but exhibited significant bias under the *R_Hu_* < 0.36 condition when compared to ICA.

**Figure 3:**
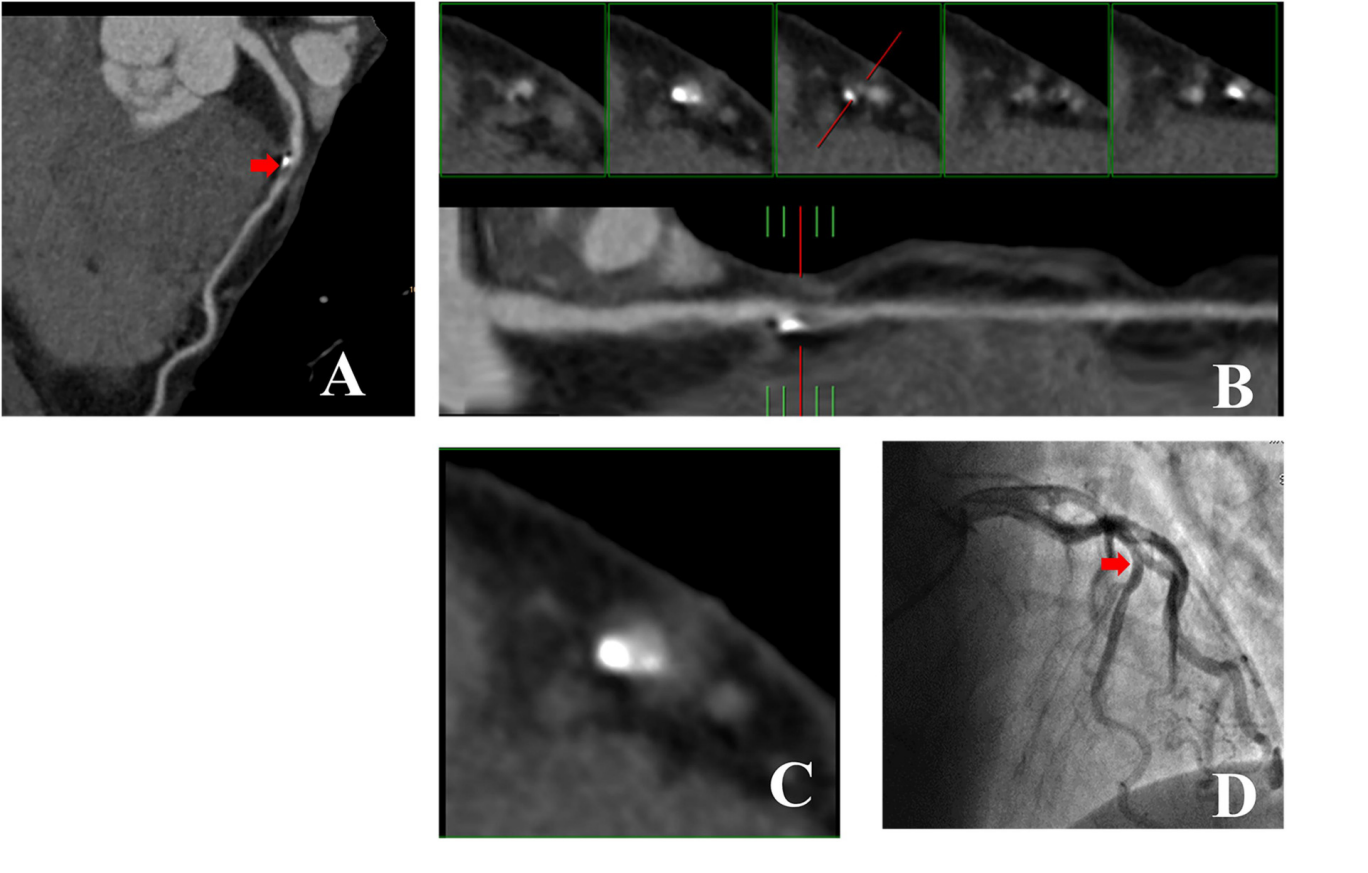
53-year-old man with R*_Hu_* = 0.30 which CCTA images showed large calcified plaque in LAD artery was no corresponded to ICA. **(A)** Curved MPR image of LAD artery shows large calcified plaque proximally. **(B)** Straightened view of LAD artery lumen. The red line in lower panel indicate the cursor, which can be moved along length of vessel by mouse. The interval of the five small panels above was 2 mm. Middle of the panels shows cross section of vessel at location indicated by cursor. Left two small panels show cross sections proximal to cursor location, while two small panels to right show cross sections distal to it. Calcified plaque occupies entire central portion of lumen and was correctly interpreted to be stenosis lesion. **(C)** Enlarged view of middle small panel showed in **(B)**. **(D)** ICA image of left coronary artery in right anterior oblique projection shows no obvious LAD artery stenosis at the position demarcated by cursor in (**B,C)**. CCTA, coronary computed tomography angiography; ICA, invasive coronary angiography; R_*Hu*_, ratio of HU values between calcified plaque and blood; MPR, curved multiplanar reformation; LAD, Left anterior descending.

**Figure 4:**
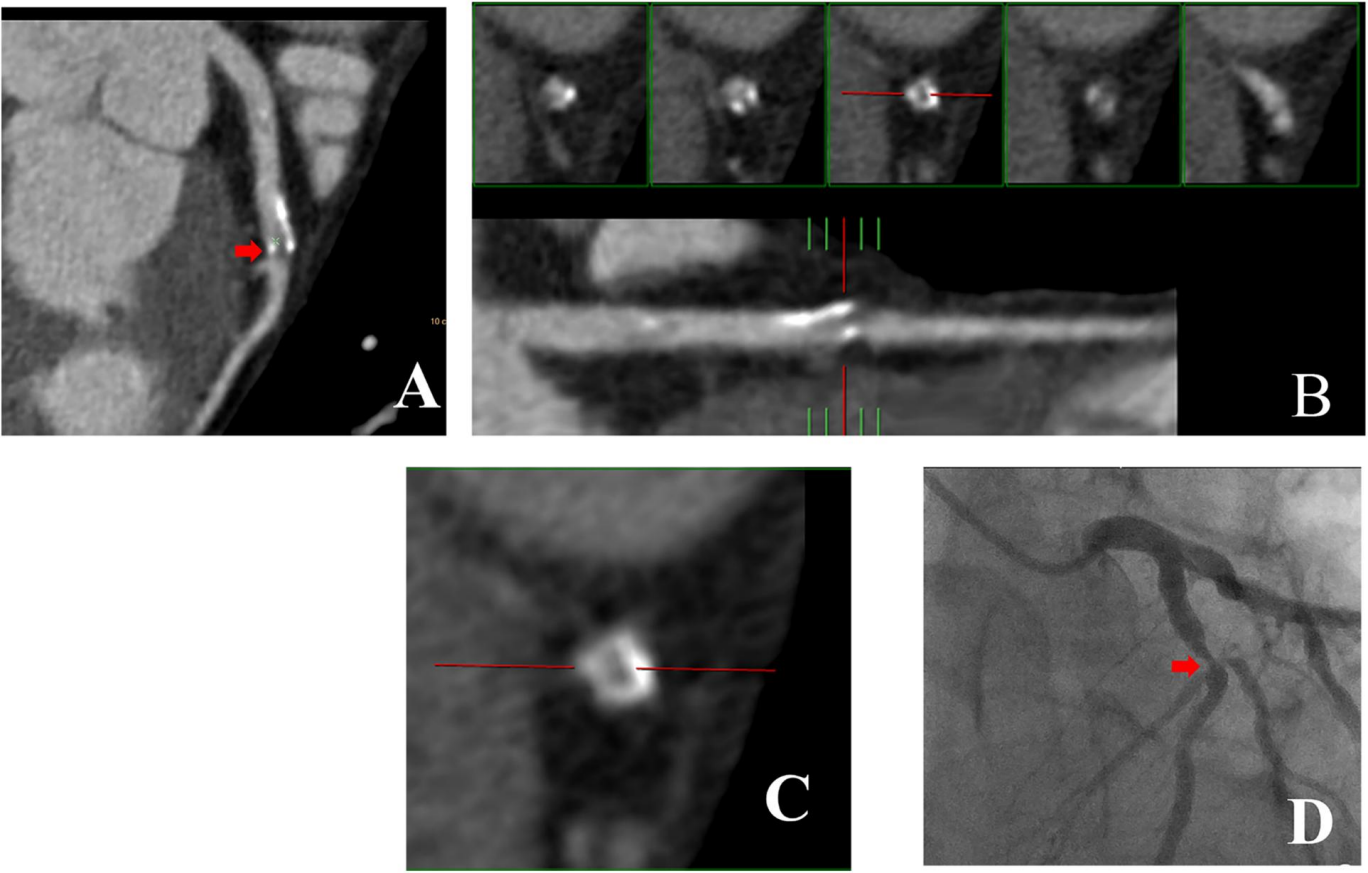
43-year-old man with R*_Hu_* = 0.38 which CCTA images shown large calcified plaque in LAD artery was corresponded to ICA. **(A)** Curved MPR image of LAD artery shows large calcified plaque proximally. **(B)** Straightened view of LAD artery lumen. **(C)** Enlarged view of middle small panel shown in **(B)**. **(D)** ICA image of coronary artery in right anterior oblique projection shows obvious LAD artery stenosis at the position demarcated by cursor in (**B**,**C)**. CCTA, coronary computed tomography angiography; ICA, invasive coronary angiography; R_*Hu*_, ratio of HU values between calcified plaque and blood; MPR, curved multiplanar reformation; LAD, Left anterior descending.

Figures 3,4 illustrate examples of calcified plaques as observed in CCTA alongside the corresponding ICA images for two distinct conditions. Specifically, Figures 3A,4A present curved MPR images of the left anterior descending (LAD) artery, which contains a large calcified plaque. Figures 3B,C,4B,C depict a straightened view of the lumen and cross-sectional images of the vessel at the site where the calcified plaque most significantly encroaches upon the lumen. Additionally, Figures 3D,4D display the corresponding ICA images, which indicate whether stenosis resulting from the calcified plaque in the LAD artery occurred.

### 3.3 *In vitro* experiment

For the Hu values of the outer tube (“vessel”) and inner tube (“plaque”), we designed two experiments in which one CT number was fixed while the other was varied. In both experiments, the ratios increased, and the diameter measurement was closer to the true diameter (2 mm). In addition, the results also revealed that at a ratio exceeding 0.36, the diameter measurement was reliable (Supplementary Figure S5).

### 3.4 Validation experiment

The *R_Hu_* demonstrated a prediction accuracy of 74.0% in the validation cohort. The PPV and NPV were 60.0% and 87.5%, respectively. The peak frequency occurred at *R_Hu_* = 0.364 or *R_Hu_* = 0.295 in the patients whose CCTA results were consistent or inconsistent with the ICA results, respectively (Supplementary Figure S6).

## 4 Discussion

Advances in CT technology, particularly in temporal and spatial resolution, have significantly enhanced the robustness and accuracy of CCTA for the non-invasive assessment of CAD. These enhancements are primarily attributed to reductions in image noise, improvements in diagnostic image quality, and minimized blooming artifacts. Numerous studies have compared the ability of CCTA in the evaluation of coronary artery stenosis caused by calcified plaques against coronary catheterization or intravascular ultrasound (IVUS) (14, 26, 27). Our study utilized a considerable sample size to suggest that assessing the difference in Hu values between blood and adjacent calcified plaques can effectively gauge the accuracy of CCTA for stenosis detection in patients with calcified coronary arteries.

Although single-energy computed tomography (SECT) is considered as the gold standard imaging modality for identifying calcification, the overlap of calcified plaque in radiodensities precludes accurate material composition assessment (28, 29). In general, if the SECT attenuation level of a lesion is below 100 Hu, it was considered as calcification (30). Numerous studies have identified that calcification is the major cause underlying the overestimation of luminal stenosis by CCTA and is the sole factor impacting the diagnostic accuracy of CCTA in comparison to ICA (13, 31). Blooming artifacts can exaggerate the size of calcified plaques and diminish the accuracy of the evaluation of the adjacent coronary artery lumen, resulting in an overestimation of lesion severity (32). Research has demonstrated that the implementation of environmental interventions, such as music and visual stimuli, in the waiting area for CCTA can effectively reduce anxiety and heart rate. This reduction may subsequently lead to a decrease in the use of radiation and beta-blockers, thereby indirectly enhancing image quality (33). Our studies suggest that under specific conditions, the degree of stenosis derived from CCTA can be reliable, thereby enhancing the efficiency of the CCTA process and simplifying the protocol by avoiding unnecessary complex algorithmic calculations.

The 2017 Clinical Guideline of National Institute for Health and Care Excellences (NICE) suggested that detection of large (≥ 50%) stenosis derived from non-invasive modalities was the reference standard of clinical decision-making for cardiac catheterization (34). In a multicenter trial, the NPV of CCTA for the detection of coronary diameter stenosis greater than 50% was 99% (35). In our study, we speculated that the *R_Hu_* could serve as a predictive parameter to assess the accuracy of the CCTA in detecting ≥50% stenosis. In practice, we used the ICA results as the golden standard of stenosis detection, and categorize 50% stenosis as a threshold value in the two-dimensional ROC analysis of *R_Hu_*. This conclusion is further supported by the AUC values (0.789). Additionally, Spearman's correlation coefficient for the percentage of diameter stenosis vs. *R_Hu_* corroborates this finding. The increasing calcium burden is associated with reduced CCTA diagnostic accuracy, which is consistent with the findings of the cited 2024 study comparing CCTA and ICA (36). Our stratified results support this trend, indicating that when the Agatston scores ≥1,000, sensitivity reaches 100% while specificity drops to 52.9%. Notably, although specificity decreases slightly, the R*_Hu_* retains its effective discriminatory capability.

The Hu scale in a voxel is proportional to the linear attenuation coefficient μ(E), which represents the rate of attenuation of a monoenergetic beam as it passes through a given material. This coefficient is influenced by the chemical composition, the photon energies interacting with the object, and the mass density of the material (37, 38). An increase in the iodine delivery rate (IDR), in conjunction with the x-ray tube voltage, will alter the Hu value of blood (39). Compelling evidence suggests that during the dynamics of cardiovascular contrast medium, increasing the IDR through faster injection flow rates and/or a higher iodine concentration in the contrast medium (CM) results in a diminished enhancement magnitude of blood, evidenced by a decrease in the Hu value (40). However, the reduction in Hu value associated with CM concentration has minimal impact on high-atomic-number materials (e.g., calcified plaque) (41). Therefore, it is feasible to adjust the Hu value by manipulating the IDR and/or utilizing higher concentrations or volumes of contrast media (39). Nevertheless, there is growing concern regarding increased radiation exposure in clinical practice, as it can elevate the renal burden, particularly in the elderly population and individuals with underlying diseases and risk factors. In the *in vivo* experiment conducted in our study, the radiation dose required to adjust the IDR to make *R_Hu_* exceed 0.36 was <14 mSv, which does not exceed the recommended safety dose as outlined in the American College of Radiology (ACR) white paper on radiation dose in medicine (42). In a previous in-house study involving a sample size of 120,822, they found that the incidence of adverse drug reactions following non-ionic iodinated contrast media (NICM) delivery was extremely low in patients, irrespective of their underlying conditions (43). The total dose of iohexol-350 administered was approximately 0.8–1.0 ml/kg of body weight, which is well below the FDA-recommended dose for elderly patients with pre-existing renal disease. Furthermore, considering that this method may avoid unnecessary ICA examinations, the total radiation dose can be significantly reduced.

As a pilot study, our research is a single-center investigation focusing on a specific cohort. It is important to note that the difference in patient conditions and intrascanner variation in CT number measurements was not evaluated. Previous reports indicate that measured CT numbers exhibit variability among manufacturers (28). Another limitation of our study is the absence of an intrinsically different imaging modality (such as IVUS) as an external reference standard to validate our volumetric measurements of coronary artery calcifications. Given our retrospective design, invasive intravascular imaging was unavailable in most patients. We have added a prospective plan to compare R*_Hu_* against IVUS/OCT-derived lumen area and calcium arc/length in future work. Additionally, the conduction of CTA without nitroglycerin, which is strongly recommended to enhance both image quality and diagnostic confidence, represents another limitation.

## 5 Conclusions

In summary, our study indicates that the Hu difference (R*_Hu_*) between blood and calcified plaques is a significant factor influencing the detection of stenotic vessels with calcified plaques using CCTA, with a cutoff value of 0.36. The R_Hu_ analysis is expected to enhance the effectiveness of CCTA in supporting clinical decision-making for ICA in patients with CAD. We also plan to conduct follow-up multi-center studies to further validate our findings.

## Data Availability

The raw data supporting the conclusions of this article will be made available by the authors, without undue reservation.
