# Supplementary material for: When can coronary computed tomography angiography in patients with calcified plaque be accurate?
Source: Front Cardiovasc Med. 2025 Sep 22;12:1570517. doi: 10.3389/fcvm.2025.1570517 (PMC12497761; doi:10.3389/fcvm.2025.1570517)
Supplement: Supplementary file 1 [file Datasheet1.pdf]

Supplementary Materials

Inclusion and exclusion criteria

The inclusion criteria were adopted as follows: (1) Assessment by a cardiologist indicating a diagnosis of CAD; (2) The patient is 18 years or older; (3) The interval between CCTA and ICA examinations must not exceed 14 days; (4) Complete clinical data must be available. Exclusion criteria include: (1) Blurred CCTA images or significant discontinuity or motion artifacts; (2) Severe artifacts present in the superior vena cava; (3) Only soft plaques observed; (4) Instances of mixed plaques in which the calcified component occupies less than 50% of the plaque arc length or less than 50% of the plaque attenuation voxels; (5) A history of prior stent implantation or bypass grafting; (6) Concurrent severe renal insufficiency, end-stage cancer, complex heart disease, etc.; (7) Allergy to iodine contrast agents.

Measurement Methods for Coronary Artery Stenosis in CCTA

Referencing the curved planar and axial images of CCTA, the lesion diameter was measured at the maximum level of the calcified plaque and recorded as $D_{max\ plaque}$. Considering that the vessel at the site of the calcified plaque is often deformed due to mass effect, we measured the vessel diameter at both ends near the calcified plaque lesion, recorded as D_{blood1} and D_{blood2}. The average of these two values was recorded as D_{blood} (Fig. S1). The degree of stenosis at the coronary plaque site according to the following formula:

$$\%Stenosis = \frac{D_{blood} - D_{max\,plaque}}{D_{blood}}$$

Incremental Clinical Utility Assessment

Two nested logistic regression models were developed for incremental analysis. The base model (CCTA model) included CCTA-derived stenosis severity as the sole predictor. The augmented model (CCTA + R_{Hu} model) incorporated R_{Hu} values in addition to CCTA stenosis. Predicted probabilities from both models were subsequently used for comparative evaluation of discrimination, reclassification, decision curve analysis (DCA), and calibration performance. All advanced statistical analyses were performed using R software (version 4.3.2) with the 'PredictABEL' and 'rms' packages.

To evaluate the incremental clinical value of R_{Hu}, additional statistical analyses were performed. The net clinical benefit of adding R_{Hu} to CCTA was assessed using DCA, where net benefit was calculated across a range of threshold probabilities according to the standard formula:

$$Net\ Benefit = \frac{TP}{N} - \frac{FP}{N} \times \frac{threshold}{1 - threshold}$$

To improve probability distribution stability, the predicted probabilities derived from logistic models were transformed using a 4th root compression prior to DCA. The net benefit curves were smoothed using a moving average window to minimize potential fluctuation artifacts.

Reclassification analyses including net reclassification index (NRI) and integrated discrimination improvement (IDI) were further performed to quantify the reclassification ability of R_{Hu}.

Finally, model calibration was assessed using bootstrap resampling (B=1000 iterations), generating calibration plots including apparent, bias-corrected, and ideal curves to visualize the agreement between predicted and observed probabilities.

Incremental Diagnostic Value of R_{Hu}

The NRI was 0.5, entirely driven by improved correct reclassification among non-events (NRI_{event} = 0; $NRI_{nonevent}$ = 0.5), indicating that the addition of R_{Hu} mainly contributed to improved identification of patients without obstructive CAD. The IDI was 0.248, reflecting substantial overall enhancement in model discrimination after incorporating R_{Hu}.

DCA (Fig. S3) demonstrated that the model incorporating R_{Hu} consistently provided higher net benefit than CCTA alone across high threshold probabilities (> 90%), suggesting superior clinical utility in correctly identifying patients with obstructive lesions while minimizing unnecessary downstream testing.

Calibration analysis (Fig. S4) revealed good agreement between predicted and observed risks. The bias-corrected calibration curve closely followed the ideal diagonal line, supporting the robustness and calibration accuracy of the prediction model including R_{Hu}.

Supplementary Figures

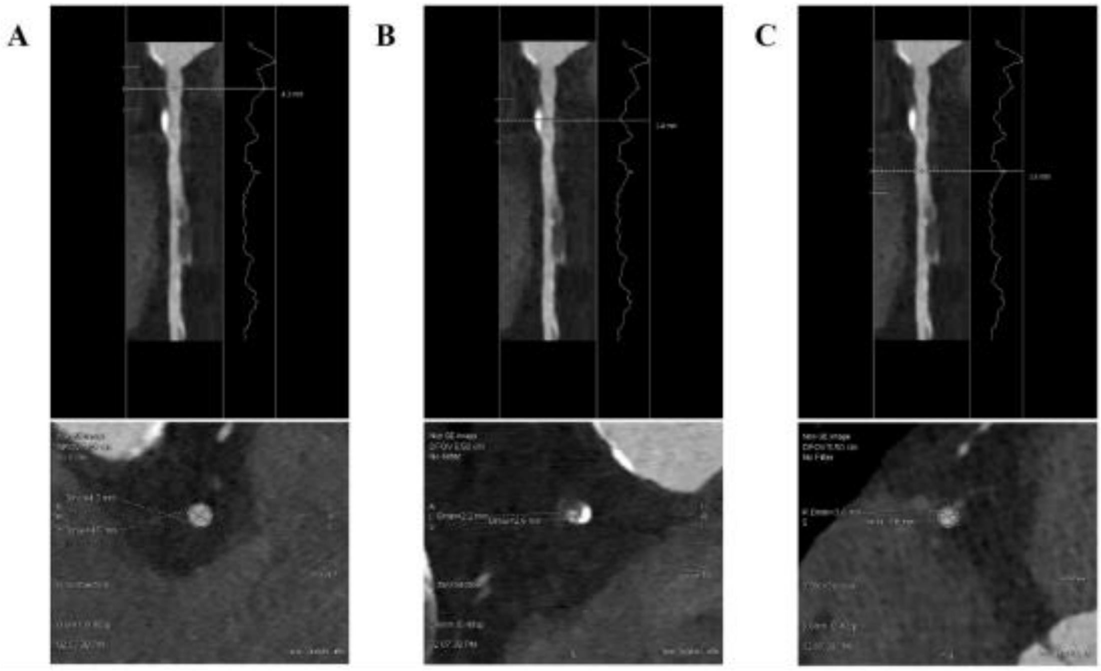

Figure S1. The measurement steps for coronary artery stenosis in CCTA. **A**, Diameter of blood ROI 1. **B**, Diameter of stenoses lesion. **C**, Diameter of blood ROI 2. CCTA, coronary computed tomography angiography; ROI, region of interest.

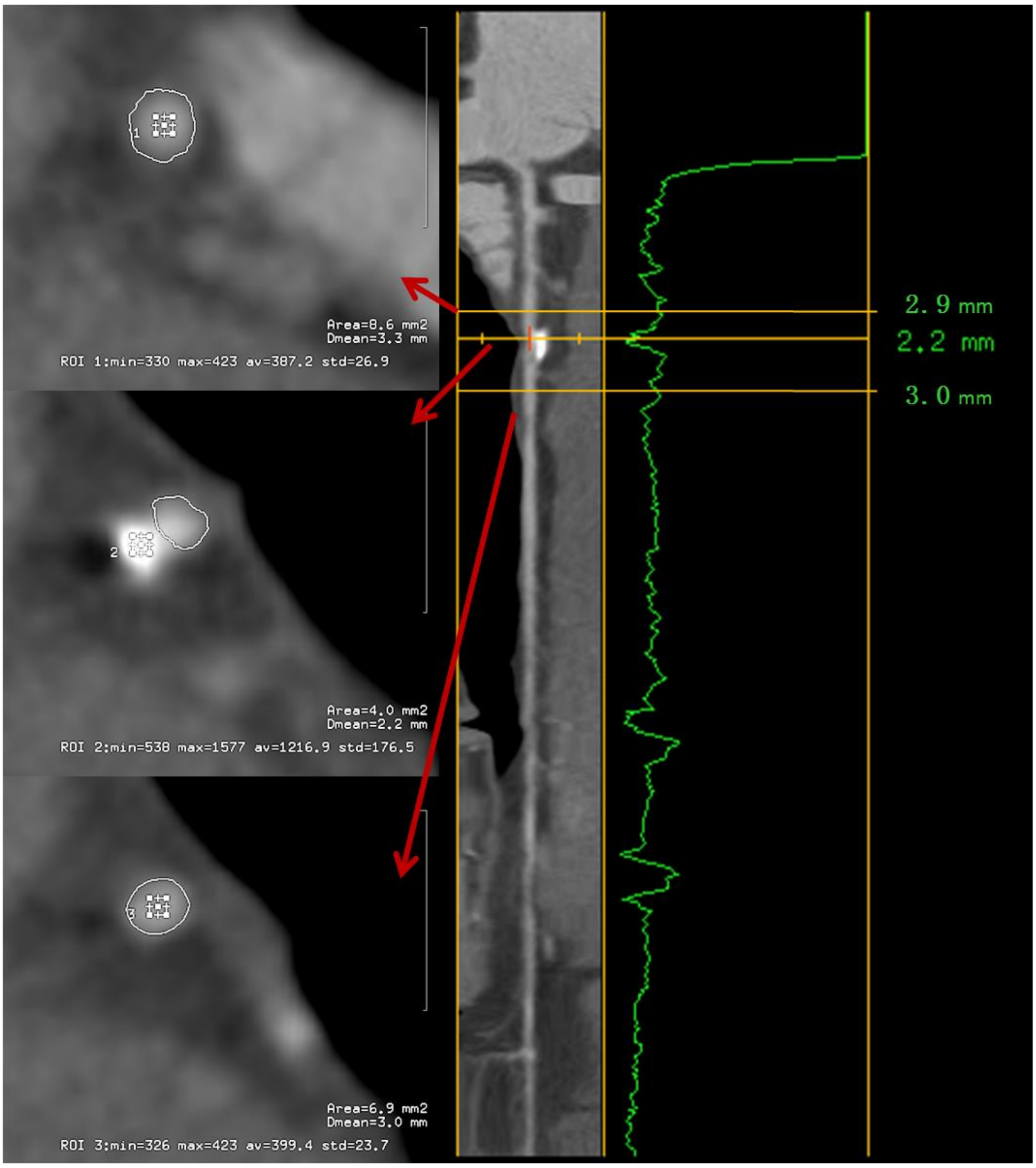

Figure S2. The measurement of HU values in both the plaque and blood. The HU value of the blood was measured in the near -heart of the plaque (ROI 1), while the HU value of the plaque was assessed at the maximum level (ROI 2). HU, Hounsfield unit; ROI, region of interest.

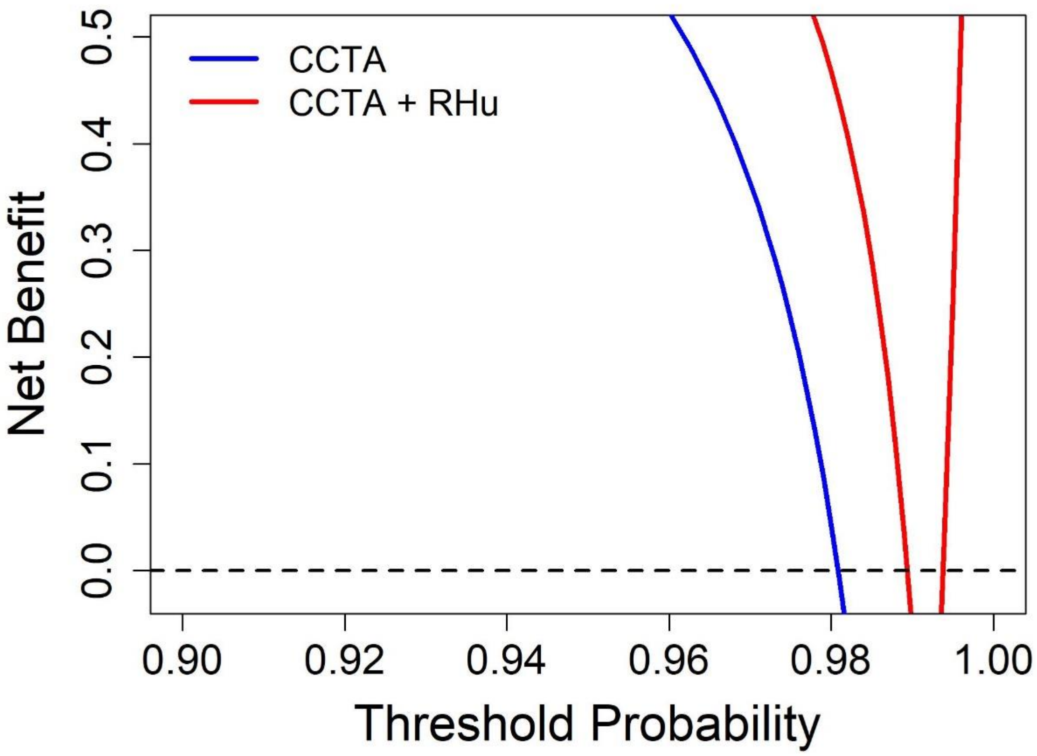

Figure S3. DCA for evaluating the incremental clinical utility of R_{Hu}. The blue curve represents CCTA alone, and the red curve represents CCTA combined with R_{Hu}. The model incorporating R_{Hu} yields higher net benefits across high threshold probabilities (> 90%), suggesting improved clinical usefulness in identifying patients with obstructive coronary lesions while reducing unnecessary interventions. DCA, decision curve analysis; CCTA, coronary computed tomography angiography; R_{Hu}, ratio of HU values between calcified plaque and blood.

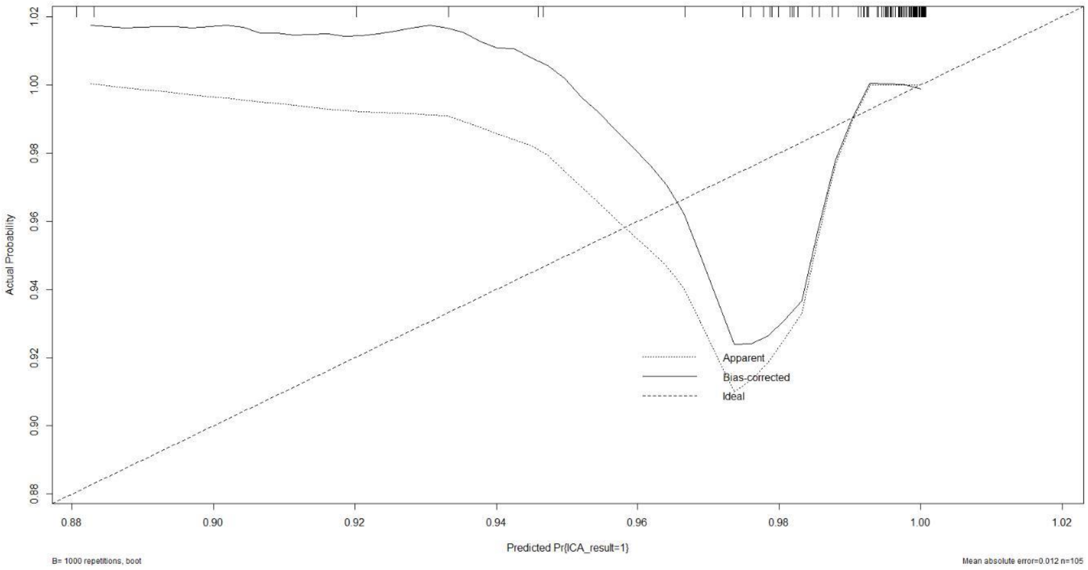

Figure S4. Calibration plot for the CCTA + R_{Hu} model. The solid line represents the bias-corrected curve obtained by bootstrap resampling (1000 repetitions), the dotted line indicates the apparent calibration, and the dashed diagonal line represents perfect calibration. The close proximity of the bias-corrected curve to the ideal diagonal indicates good agreement between predicted and observed probabilities. CCTA, coronary computed tomography angiography; ICA, invasive coronary angiography; R_{Hu}, ratio of HU values between calcified plaque and blood.

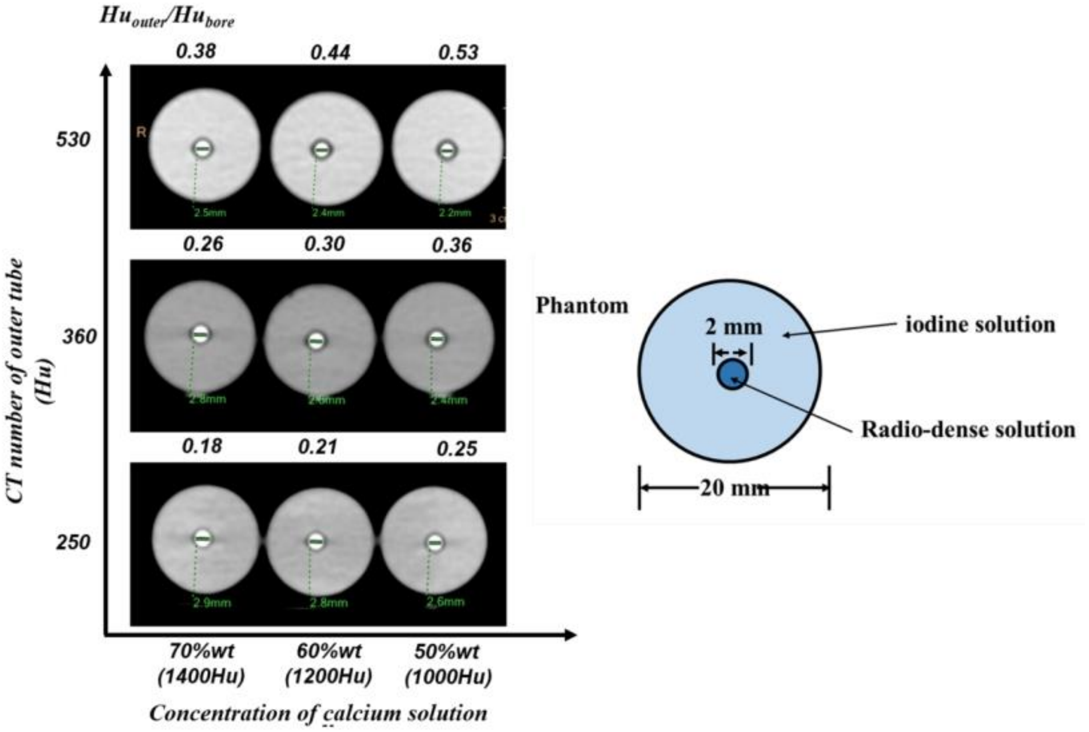

Figure S5. In vitro experiments. The left panel was the results of CT scan of the phantom. The vertical axis represents CT number of the out tube, while the horizontal axis represents concentration of concentration of calcium solution in inner tube. The right panel was the sketch of phantom. Hu, Hounsfield units; CT, computed tomography.

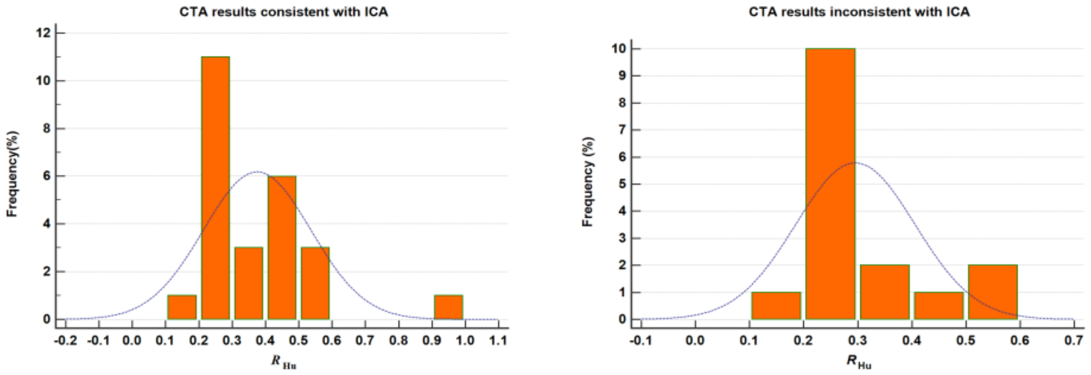

Figure S6. Generation of R_{Hu} histograms in validation cohort. CTA, computed tomography angiography; ICA, invasive coronary angiography; Hu, Hounsfield units;

R_{Hu}, ratio of HU values between calcified plaque and blood.
